# Tau as a fluid biomarker of concussion and neurodegeneration

**DOI:** 10.2217/cnc-2022-0004

**Published:** 2022-12-06

**Authors:** Iftakher Hossain, Kaj Blennow, Jussi P Posti, Henrik Zetterberg

**Affiliations:** 1 Department of Neurosurgery, Neurocenter, Turku University Hospital, Finland; 2 Turku Brain Injury Center, Turku University Hospital, Finland; 3 Department of Clinical Neurosciences, University of Turku, Finland; 4 Department of Clinical Neurosciences, Neurosurgery Unit, University of Cambridge, Addenbrooke's Hospital, Cambridge, UK; 5 Institute of Neuroscience & Physiology, Department of Psychiatry & Neurochemistry, The Sahlgrenska Academy at the University of Gothenburg, Mölndal, Sweden; 6 Clinical Neurochemistry Laboratory, Sahlgrenska University Hospital, Mölndal, Sweden; 7 Department of Neurodegenerative Disease, UCL Institute of Neurology, Queen Square, London, UK; 8 UK Dementia Research Institute at UCL, University College London, London, UK; 9 Hong Kong Center for Neurodegenerative Diseases, Hong Kong, China

**Keywords:** chronic traumatic encephalopathy, concussion, mild traumatic brain injury, neurodegenerative disease, Tau

## Abstract

Concussion is predominant among the vast number of traumatic brain injuries that occur worldwide. Difficulties in timely identification, whether concussion led to neuronal injury or not, diagnosis and the lack of prognostic tools for adequate management could lead this type of brain injury to progressive neurodegenerative diseases. Tau has been extensively studied in recent years, particularly in repetitive mild traumatic brain injuries and sports-related concussions. Tauopathies, the group of neurodegenerative diseases, have also been studied with advanced functional imaging. Nevertheless, neurodegenerative diseases, such as chronic traumatic encephalopathy, are still conclusively diagnosed at autopsy. Here, we discuss the diagnostic dilemma and the relationship between concussion and neurodegenerative diseases and review the literature on tau as a promising biomarker for concussion.

## Concussion: diagnostic dilemma

More than 90% of patients who sustain traumatic brain injury (TBI) are classified in the mild end of the spectrum, including injuries referred to as concussion [1,2], which is common, for example, in contact sports [3,4]. Currently, there is no internationally agreed definition of concussion. It has been reported that mild TBI (mTBI) and concussion should be considered as distinct entities [5]. Concussion was defined as *“a complex pathophysiological process affecting the brain, induced by traumatic biomechanical forces”.* On the other hand, the current American Academy of Neurology guidelines for sports concussion do not distinguish between concussion and mTBI [6], defining concussion as* “a clinical syndrome of biomechanically induced alteration of brain function, typically affecting memory and orientation, which may involve loss of consciousness”*. In general, concussion does not cause gross pathology, such as hemorrhage, or other abnormalities on structural brain imaging [7]. Therefore, concussion usually does not cause loss of consciousness, but cause many other complaints such as dizziness, nausea, decreased attention and concentration, memory problems and headache [1,8]. mTBI and concussion are relatively similar, and the two terms are used inter-changeably in the literature. A key research and clinical question is whether the external trauma to the head, except for the symptoms linked to the clinical concussion entity, also lead to injury of neuronal or other types of brain cells (e.g., astrocytes) or structures (e.g., capillaries).

There is also no clear pathologic definition or criteria to distinguish concussion from other types of TBI, and the injuries leading to concussion are biomechanically similar to other types of TBI. TBI with biomechanical forces to the brain causes a neurometabolic cascade that affects the brain function [9–11]. The disrupted neurometabolic state can last for days and the consequences of these cascades of TBI have been described as evolving phenomena [12].

Although many patients have good recovery within weeks or months following mTBI, 5–30% of patients with mTBI suffer from neurologic, cognitive, and/or neuropsychiatric symptoms for one year post-injury or longer [1,13–15]. All of these persistent symptoms or findings are known as post-concussion syndrome (PCS), which could be defined as a collection of post-traumatic symptoms [16,17]. Even though the risk of developing PCS could be predisposed by injury mechanisms, pre-injury mental health, and socioeconomic factors, it is unclear why PCS appears, perseveres, or resolves [18,19]. Given that the standard tool for assessing acute TBI, head computed tomography (CT), is not sensitive enough to detect microbleeds and traumatic axonal injury [20], clinicians are lacking an objective test to predict the outcome of patients with mTBI or concussion. This could be helpful in stratifying the group of patients who might need observation, further treatment, follow-up and rehabilitation [21,22].

## Concussion & neurodegenerative disease

Although current literature does not generally associate neurodegenerative disease development with patients who have sustained a single mTBI [23–26], research suggests that repeated mTBIs or concussions are associated with a higher risk of neurodegenerative disease years later, particularly in those patients who do not fully recover between mTBIs [27,28]. Patients with repetitive injuries have a slower recovery after mTBI, which also increases the risk for future injury [29]. Patients with repetitive mTBI typically have persistent PCS [2], increased risk for early onset Alzheimer's disease (AD) [30], and being vulnerable to develop chronic traumatic encephalopathy (CTE) [31,32]. Among all contact sports, boxing remains the sport in which repetitive concussion is the most common, and also intrinsic to the basic principle of the sport. Furthermore, American football, ice hockey, rugby, and martial arts are other contact sports where professional participation increases the risk for repetitive concussion. For example, research conducted on Olympic boxers, Swedish ice hockey players, and retired American football players reported a significantly higher risk for memory loss and persistent cognitive impairment in players with more than one concussion compared with players without any reported concussion [29,33,34]. Additionally, blast induced concussion is common among military personnel who have been deployed to different war zones.

CTE refers to a chronic and progressive brain disorder, usually associated with either one major brain trauma or repeated milder trauma, and is characterized by various neurocognitive, psychiatric and neurological dysfunctions, such as dementia, depression, memory loss, inhibition and aggressiveness, as well as parkinsonian symptoms [19,35,36]. However, the pathology of CTE might start earlier than originally thought as signs of CTE have been found in the autopsies of adolescent athletes with repeated mTBIs [37]. mTBI could trigger the tau pathology associated with AD and CTE [38,39]. Additional features of CTE include axonal injury and neuronal loss, astrogliosis, and in a proportion of cases TAR DNA-binding protein 43 (TDP-43) pathology [38,39]. Although phosphorylation of tau is a physiological phenomenon, hyperphosphorylation and aggregation of tau into neurofibrillary tangles (NFTs) is common after TBI and other brain injuries [19]. Notably, formation of NFTs is a key feature of a group of neurodegenerative diseases known as tauopathies, including AD and CTE. Each of these neurodegenerative diseases has its unique presentation of phosphorylated tau (P-tau) species [19,39–41]. In case of AD, NFTs exhibit a progressive spread throughout all cortical areas [42–44]. On the other hand, CTE is characterized by abnormal tau pathology concentrated around small blood vessels in the depth of sulci within the cortex, especially, at the interface between white and grey matter [37,45,46]. The molecular structures of tau filaments found in AD and CTE differ from each other [29]. Studies of patients with mTBI have reported that NFTs accumulate primarily in the deep sulci of the frontal and temporal cortices and along the cortical blood vessels of individuals with CTE, and contributed significantly to the long-term development of neurodegeneration [45–48]. However, it is important to note that there are no prospective, longitudinal or epidemiological studies that provide enough evidence that CTE pathology is as progressive as other neurodegenerative diseases, for example, AD. It has been reported that a single injury of any severity could cause CTE and it could begin shortly, days, weeks, months, years or decades after exposure to neurotrauma [45]. Interestingly, it has been studied that many people with the neuropathology of CTE do not appear to have progressive tauopathy and some people with the neuropathology of CTE do not show any clinical signs or symptoms that are accountable to that pathology [49]. It has recently been demonstrated that CTE pathology could be present in people who have not sustained sub-concussive blows to the head, or multiple concussions [50].

Using an objective test, like body-fluid biomarkers, to identify the group of patients with incomplete recovery, and to ensure the appropriate management, could possibly assist to reduce the occurrence of CTE. In the following sections of this brief review, we review the literature published on one of the most promising candidate biomarkers for concussion, tau, known as a neurodegenerative biomarker. Finally, we discuss the importance of fluid biomarker development for CTE.

## Tau & concussion

Tau, a microtubule-associated protein, with a molecular weight of 48–67 kDa, abundant in unmyelinated cortical axons [39,51,52], serves as a structural element in the axonal cytoskeleton of the central nervous system (CNS) and peripheral nervous system (PNS) [53,54]. Though tau is predominantly expressed in the brain, extracranial sources exist, for example, liver, kidney and testis [55]. Earlier research interest in TBI biomarkers has focused primarily on total tau (T-tau), but P-tau and cleaved tau (C-tau) have also been studied. Tau is elevated in CSF and plasma following TBI [4,19,52].

### CSF tau

Increased CSF tau concentrations have been observed in patients with TBI, and admission CSF tau concentrations were correlated with long-term outcome among these patients [56,57]. A study of CSF samples of Olympic boxers 1–6 days after bouts compared with CSF samples of healthy controls reported significant elevation of tau in the boxers' CSF [58]. Increased concentrations of CSF tau were also found in Olympic boxers after a bout, which did not necessarily correlate with the number of hits to the head [59]. Another study on college football players found a similar result, with no correlation between the number of mTBIs or concussions and the concentrations of tau, although increased plasma tau concentrations were observed after training [60]. In chronic neurodegenerative diseases, such as AD, CSF T-tau concentrations correlate poorly with plasma T-tau concentrations [61], but in acute TBI, the correlation is likely tighter, depending on the severity of injury and thus increase of T-tau from neurons to both CSF and plasma. Tau elevations following TBI could stay much longer in CSF (weeks) compared with blood (days) [29].

### Plasma tau

Since the concentrations are very low in the peripheral blood in health and disease, and therefore cannot be accurately measured by most immunoassays, the availability of ultrasensitive Single molecule array (Simoa) technology allows for the adequate quantification of T-tau in both plasma and serum [62,63]. Several studies have reported increased concentration of plasma T-tau in the context of TBI. Persistent high tau concentrations have been reported after TBI, but generally tau concentrations peak between 12–24 hours [34,64,65]. The current literature suggests that increase of tau following TBI could follow both an acute and chronic course. While the initial increase in tau concentrations indicates acute neuronal injury, the secondary increase most possibly reflects secondary pathology associated with chronic neurodegenerative processes [39,66]. Additionally, tau concentrations in blood increase with age [67,68] and a distinct temporal profile and substantially higher T-tau concentrations have recently been reported in female athletes with concussion, compared with their male counterparts [69]. Significant correlations between serum tau concentrations and neurological outcome has been reported in resuscitated cardiac arrest patients [65]. Plasma T-tau concentrations were increased 1 h after injury compared with the pre-season concentrations, and predicted return-to-play (RTP) time with significant accuracy in studies of professional ice hockey players with concussion [70,71]. Another study in concussed athletes reported that plasma T-tau concentrations at 6 h after injury significantly correlated with RTP time [72]. Increased plasma tau concentrations have been reported in military personnel exposed to blast injuries in the preceding 18 months [73]. Higher exosomal tau concentrations have also been reported to be associated with chronic symptoms in military personnel after mTBI [74]. In another study including TBIs of all severities, plasma T-tau concentrations could discriminate mTBI from controls when the samples were collected within 24 hours after injury [75]. Serum tau concentrations were also reported as a significant outcome predictor following TBI [76]. It has been recently studied that acute plasma P-tau concentrations and the P-tau-T-tau ratio outperformed T-tau concentrations for the outcome prediction of TBI [64]. Nevertheless, plasma T-tau concentrations at admission were unable to discriminate between incomplete and complete recovery in case of single and uncomplicated mTBI [63]. Contradictory results have been reported regarding the use of C-tau as a fluid biomarker as an acute biochemical diagnostic tool of mTBI [77–79].

## CTE & abnormal tau pathology

In a recent post-mortem study, the brains of deceased American football players were examined [80]. At the autopsy, CTE was diagnosed in 88% of the subjects, and the severity of the condition was significantly higher in subjects who played professional football than in subjects who only played in high school. Another alarming finding of this study is that the majority of the subjects with CTE findings had PCS and later clinical signs of dementia [80]. This suggests that repeated mTBIs or concussions earlier in life could predispose to neuropathology later in life. Utilizing positron emission tomography (PET) ligands for tau in the brain to explore the molecular pathology of CTE, it was found that retired National Football League players with a history of concussion had higher overall signals of tau deposition compared with those without concussion [81]. In a recent study [82], young patients with symptomatic repetitive sports-related concussions were found to have increased tau aggregation at longer time points after injury. To detect abnormal tau pathology, dual PET tracers in combination with biomarkers (CSF and plasma tau), neuropsychological evaluation, and 3 Tesla magnetic resonance (3T MR) scanning were used in this study [82]. These abovementioned studies and the current literature support the idea that tau pathology might have a mechanistic link to cognitive symptoms in case of repeated concussion [83,84], and the reported tau aggregation could be progressive [38], which ultimately play the critical role in the disease mechanism of neurodegenerative disorders such as CTE.

## Conclusion

Although the current studies showed several promising aspects of tau as a CSF and blood biomarker of concussion, there is still insufficient evidence to support the clinical validity for the bench-to-bedside application of this neurodegenerative biomarker [79].

## Future perspective

Since most of the current biomarker studies focused on the short-term consequences of mTBI, further large cohort studies should examine the long-term effects of repeated concussions, not only in high-impact contact sports, but also in mTBIs that are not sports-related. Moreover, brain-derived exosomes may represent new diagnostic tools in the coming future [41]. Novel assays measuring CNS-specific forms of tau (i.e., assays that do not measure peripheral, so-called big tau) are under development, and will be interesting to examine in the context of acute and chronic TBI.

**Executive summary**

- In the current literature, there is no internationally agreed definition of concussion, and the two terms concussion and mild traumatic brain injuries (mTBI) are used inter-changeably.
- A significant portion of patients with mTBI suffer from post-concussion syndrome 1 year post-injury or longer and patients with repetitive mTBI or concussion are more at risk to develop persistent post-concussion syndrome.
- Early onset of neurodegenerative diseases has been reported for the players participating in various contact sports, notably in boxing, where repetitive concussion is common.
- Chronic traumatic encephalopathy (CTE), known as a chronic and progressive disorder of brain, is associated with neurofibrillary tangle accumulation in the brain reflecting abnormal tau pathology and a proportion of cases show TDP-43 pathology. Nevertheless, there is no class one evidence stating that CTE is as progressive as other neurodegenerative diseases. Additionally, people with no history of multiple concussions have been also reported for CTE pathology.
- Body fluid biomarkers could help to stratify those patients better who are at risk of incomplete recovery. Tau, an axonal biomarker, has been extensively studied recent years in the field of mTBI and sports concussion.
- Recent studies combining advanced imaging and CSF and plasma tau reported the concept that tau pathology might have a mechanistic link to cognitive symptoms in case of repeated concussion. In addition, progressive tau aggregation might play a vital role in the disease mechanism of neurodegenerative diseases.
- Further large cohort studies implementing novel assays measuring CNS-specific forms of tau should examine the long-term effects of repeated concussions.
